# Physiology of lived experience: The struggles of my first experience of sleep at 3810 m

**DOI:** 10.1113/EP093948

**Published:** 2026-10-07

**Authors:** Julian C. Bommarito

**Affiliations:** ^1^ Human Cardiovascular Physiology Laboratory University of Guelph Ontario Canada

Sleep during our recent WM:S25 high‐altitude research expedition at the Barcroft White Mountain Research Station (∼3810 m above sea level) did not feel restorative and did not provide meaningful recovery. When a lowlander ascends to high altitude, acute sleep disturbances are common above ∼2500 m due to the hypobaric hypoxia, but the mechanisms are not fully understood. One contributing mechanism can be the accompanying reductions in nocturnal peripheral blood oxygen saturation ($S_{pO_{2}}$), which promote a phenomenon called periodic breathing, characterised by cyclic central apnoeas interspersed with periods of hyperventilation (Dempsey, 2019). Another factor that may transiently contribute to sleep impairment is the development of acute mountain sickness (AMS). Prior work has shown that AMS rates after rapid ascent to White Mountain Research Station can be as high as 73% (Lipman et al., 2018). The good news is that sleep can partially stabilise within a few days (Bloch et al., 2015). Unfortunately, this is not always the case (Bloch et al., 2015). I was excited to find out how I would fare before our travels, but that quickly changed. Even though I did not develop AMS, the first couple of nights of sleep felt non‐restorative and this pattern persisted throughout our stay, causing my mental well‐being to progressively deteriorate. What can be a transient physiological disturbance became my reality for nearly 2 weeks.

Our research group measured sleep nightly for two weeks before and throughout the 11‐night high‐altitude expedition using an FDA 510(k) cleared Class II ventilatory effort recorder (SleepImage by MyCardio LLC, provided by Empower Sleep, Los Angeles, CA, USA), worn as a finger ring. This device uses photoplethysmography to measure $S_{pO_{2}}$ and estimate heart rate and respiratory signals. Sleep staging is estimated using cardiopulmonary coupling analysis of heart rate variability and tidal volume variability, with frequency bands used to identify stable non‐rapid‐eye‐movement sleep, unstable non‐rapid‐eye‐movement sleep and rapid‐eye‐movement sleep (Thomas et al., 2005). Stable non‐rapid‐eye‐movement sleep is a marker of restorative sleep and can be characterised by stable breathing and high vagal tone. Unstable non‐rapid‐eye‐movement sleep is a marker of autonomic arousals and can be characterised by fluctuating breathing patterns and cyclic variations in heart rate. Although my sleep duration decreased modestly from baseline (∼334 m above sea level) to altitude, the greatest impact on my day‐to‐day well‐being likely stemmed from drastic changes in sleep stability. Night after night stable non‐rapid‐eye‐movement sleep completely eluded me and unstable non‐rapid‐eye‐movement sleep predominated.

The persistence of my non‐restorative sleep raised several important questions and sparked personal curiosity regarding inter‐individual variability and my own response to chronic hypobaric hypoxia. One potential explanation is heightened ventilatory control instability, or increased loop gain, which is known to exacerbate periodic breathing at high altitude. Individuals with a more sensitive chemoreflex response to hypoxia and CO_2_ fluctuations may be particularly susceptible to cyclical overcorrections in ventilation, further increasing central apnoeas and sleep fragmentation (Dempsey, 2019). Additionally, altitude‐induced hypocapnia and an exaggerated chemoreflex drive may have further narrowed the margin between eupnoea and the apnoeic threshold (Dempsey, 2019). At the same time, circadian reductions in hypercapnic ventilatory response slope during the biological night (Spengler et al., 2000) may further impair ventilatory stability around this threshold, contributing to a predominance of unstable non‐rapid‐eye‐movement sleep. This may explain why my apnoea–hypopnoea index (AHI) shifted from mild at baseline to severe every night at altitude and nearly reached triple digits some nights. However, prior work has shown that sleep can partially stabilise within 3 days of high‐altitude exposure (∼4559 m) despite further increases in periodic breathing, with increases in slow‐wave sleep associated with increases in nocturnal $S_{pO_{2}}$ (Nussbaumer‐Ochsner et al., 2012). This suggests hypoxaemia, independent of the severity of periodic breathing, could contribute to impaired sleep quality at high altitude. Interestingly, hypobaric hypoxia equivalent to 12,000 ft or ∼3658 m can disrupt the circadian system, and this disruption is linked to impaired sleep quality (Coste et al., 2004). I am someone whose sleep–wake schedule changes quite a bit from weekdays to weekends. It is possible that an individual with an already disrupted circadian system, as seen through chronic social jet lag (i.e., the difference between the midpoint between sleep and wake time on workdays and free days), is more susceptible to sleep disturbances at high altitude. This can be a research question for future work.

Another potential mechanism contributing to my persistent non‐restorative sleep may be pre‐existing insomnia symptoms. For as long as I can remember, I have had trouble maintaining sleep, and could be characterised as having insomnia symptoms (Crawford et al., 2017). Insomnia has been associated with elevated cortisol, a potent promoter of wakefulness (Passos et al., 2023). High‐altitude exposure itself is also known to increase cortisol concentrations in lowlanders. For example, Park et al. (2014) found that salivary morning cortisol levels were higher at 3540 and 3800 m while nocturnal cortisol concentrations were higher at 3800 m compared to 1100 m. This raises the possibility that high altitude exacerbated an already dysregulated arousal system. The influence of insomnia on sleep acclimatisation to chronic hypobaric hypoxia is an interesting research area currently unexplored. It should also be noted that during the first half of our expedition, I was sleeping in a small and cold bed and in a room full of other co‐investigators, some of whom (who will remain nameless) snore loudly. However, during the second half of the expedition, I was sleeping in a larger, more comfortable bed, distanced from my peers, yet my symptoms persisted, indicating that the sleeping environment had little influence on these disturbances. In fact, my sleep duration decreased and my AHI increased during my more ‘comfortable’ sleep.

This type of research has real‐world implications. For example, many individuals travel to, or work at, high altitude and may expect sleep disruption to be transient and resolved with acclimatisation. However, my experience and prior research indicate that this may not be the case for everyone (Bloch et al., 2015). Additionally, about 16.2% of adults have insomnia globally (Benjafield et al., 2025) and 19.3% of US adults experience chronic heavy social jet lag, defined as a difference of 2 h or more (Di et al., 2022). This raises the question of whether these populations are more susceptible to sustained non‐restorative sleep at high altitudes. Persistent unstable sleep at high altitude may have downstream effects on overall well‐being, particularly during prolonged stays. Generally, I am a social person who enjoys human connection. However, over the course of the 11‐night expedition, I progressively became more irritable and isolated from the group, coinciding with persistent non‐restorative sleep. Potential strategies to mitigate high‐altitude sleep disturbances therefore warrant consideration. A highly studied remedy for impaired sleep quality at high altitude is acetazolamide, which inhibits carbonic anhydrase to induce metabolic acidosis, thereby increasing nocturnal ventilation and $S_{pO_{2}}$ resulting in reduced periodic breathing (Liu et al., 2017). Due to the confounding effects of acetazolamide on some of the studies being run, I did not take acetazolamide at high altitude. Otherwise, a 250 mg daily dose of acetazolamide could be an efficacious pharmacological intervention to improve sleep disturbances caused by hypobaric hypoxia (Liu et al., 2017), although its use is prohibited under World Anti‐Doping Agency regulations. Additionally, ascent rate to high altitude can predict AMS, with faster ascent increasing the prevalence of AMS (Beidleman et al., 2019). Therefore, a slower ascent to high altitude may be a preventative strategy to mitigate transient sleep disturbances in individuals susceptible to AMS.

The research expedition taught me a lot about how my own sleep responds to ∼3810 m above sea level. My first experience at high altitude highlights that sleep resilience and adaptability during chronic exposure likely vary considerably between individuals. This highlights an important gap, as the current body of literature does not fully explain the substantial inter‐individual variability in response to high altitude. In my case, the combination of ventilatory instability, heightened arousal and pre‐existing sleep fragmentation may have created a physiological challenge my body could not adapt to, leading to persistent non‐restorative sleep. Understanding why sleep can stabilise in some individuals at high altitude but remains a persistent struggle in others is a critical next step in high‐altitude sleep research and may help inform tailored interventions.

## AUTHOR CONTRIBUTIONS

Sole author.

## CONFLICT OF INTEREST

None declared.

## GENERATIVE AI STATEMENT

The author would like to disclose the use of OpenAI (ChatGPT version 5.5) for assistance with improving clarity, sentence structure and flow. However, ideas and interpretations remain the authors.
